# Risk of Microvascular Complications in Newly Diagnosed Type 2 Diabetes Patients Using Automated Machine Learning Prediction Models

**DOI:** 10.3390/jcm13237422

**Published:** 2024-12-05

**Authors:** Amar Khamis, Fatima Abdul, Stafny Dsouza, Fatima Sulaiman, Muhammad Farooqi, Fatheya Al Awadi, Mohammed Hassanein, Fayha Salah Ahmed, Mouza Alsharhan, Ayesha AlOlama, Noorah Ali, Aaesha Abdulaziz, Alia Mohammad Rafie, Nandu Goswami, Riad Bayoumi

**Affiliations:** 1 Hamdan Bin Mohammed College of Dental Medicine, Mohammed Bin Rashid University of Medicine and Health Sciences, Dubai P.O. Box 505055, United Arab Emirates; amar.hassan@dubaihealth.ae; 2 College of Medicine, Mohammed Bin Rashid University of Medicine and Health Sciences, Dubai P.O. Box 505055, United Arab Emirates; fatima.abdul@dubaihealth.ae (F.A.); stafny.dsouza@dubaihealth.ae (S.D.); fatima.sulaiman@mbruniversity.onmicrosoft.com (F.S.); 3 Dubai Diabetes Center, Dubai Health, Dubai P.O. Box 7272, United Arab Emirates; mhfarooqi@dubaihealth.ae; 4 Endocrinology Department, Dubai Hospital, Dubai Health, Dubai P.O. Box 7272, United Arab Emirates; ffalawadi@dubaihealth.ae (F.A.A.); mmhassanein@dubaihealth.ae (M.H.); 5 Pathology and Genetics Department, Dubai Hospital, Dubai Health, Dubai P.O. Box 7272, United Arab Emirates; faaahmed@dubaihealth.ae (F.S.A.); maalsharhan@dubaihealth.ae (M.A.); 6 Primary Healthcare Centre, Dubai Health, Dubai P.O. Box 7272, United Arab Emirates; amalolama@dubaihealth.ae (A.A.); nabawady@dubaihealth.ae (N.A.); aaalali@dubaihealth.ae (A.A.); amrafie@dubaihealth.ae (A.M.R.); 7 Center for Space and Aviation Health, Mohammed Bin Rashid University of Medicine and Health Sciences, Dubai P.O. Box 505055, United Arab Emirates; nandu.goswami@dubaihealth.ae

**Keywords:** type 2 diabetes, T2D subtypes, microvascular complications, risk prediction, machine learning

## Abstract

**Background/Objectives:** In type 2 diabetes (T2D), collective damage to the eyes, kidneys, and peripheral nerves constitutes microvascular complications, which significantly affect patients’ quality of life. This study aimed to prospectively evaluate the risk of microvascular complications in newly diagnosed T2D patients in Dubai, UAE. **Methods:** Supervised automated machine learning in the Auto-Classifier model of the IBM SPSS Modeler package was used to predict microvascular complications in a training data set of 348 long-term T2D patients with complications using 24 independent variables as predictors and complications as targets. Three automated model scenarios were tested: Full All-Variable Model; Univariate-Selected Model, and Backward Stepwise Logistic Regression Model. An independent cohort of 338 newly diagnosed T2D patients with no complications was used for the model validation. **Results:** Long-term T2D patients with complications (duration = ~14.5 years) were significantly older (mean age = 56.3 ± 10.9 years) than the newly diagnosed patients without complications (duration = ~2.5 years; mean age = 48.9 ± 9.6 years). The Bayesian Network was the most reliable algorithm for predicting microvascular complications in all three scenarios with an area under the curve (AUC) of 77–87%, accuracy of 68–75%, sensitivity of 86–95%, and specificity of 53–75%. Among newly diagnosed T2D patients, 22.5% were predicted positive and 49.1% negative across all models. Logistic regression applied to the 16 significant predictors between the two sub-groups showed that BMI, HDL, adjusted for age at diagnosis of T2D, age at visit, and urine albumin explained >90% of the variation in microvascular measures. **Conclusions:** the Bayesian Network model effectively predicts microvascular complications in newly diagnosed T2D patients, highlighting the significant roles of BMI, HDL, age at diagnosis, age at visit, and urine albumin.

## 1. Introduction

In the mid-20th century, the long-term effects of diabetes began to be more widely recognized, marked by an increase in visual impairment, renal dysfunction, and neuropathy among patients [1]. These complications, which predominantly affect tissues where glucose uptake is independent of insulin activity (e.g., kidney, retina, and vascular endothelium), result from prolonged hyperglycemia-induced damage to small blood vessels [2]. Disease progression arises from a combination of direct glucose-mediated endothelial damage, oxidative stress caused by superoxide overproduction, and the formation of sorbitol and advanced glycation end-products due to the persistent hyperglycemic state [2,3]. These metabolic injuries lead to altered blood flow, changes in endothelial permeability, extravascular protein deposition, and coagulation, all contributing to organ dysfunction. Diabetic nephropathy involves glomerular basement membrane thickening, mesangial expansion, and glomerulosclerosis, ultimately impairing kidney function and progressing to end-stage renal disease. Diabetic retinopathy is characterized by damage to retinal blood vessels, leading to microaneurysms, hemorrhages, exudates, and neovascularization, which can result in vision loss. Diabetic neuropathy involves damage to peripheral nerves, causing symptoms such as pain, tingling, and numbness, primarily in the extremities [4].

Early predictions of these complications relied on simple scoring systems based on factors such as the duration of diabetes, blood sugar control, and the presence of comorbidities to identify high-risk patients. With advancements in medical informatics, researchers started employing statistical techniques such as logistic regression, enabling the analysis of large datasets and revealing more complex relationships between various factors and the risk of complications, as exemplified by the Framingham Heart Study [5]. The focus then shifted toward multifactorial models that considered genetics and lifestyle habits, paving the way for tailored treatment approaches. Today, the rise of machine learning algorithms, such as artificial neural networks, offers a new level of sophistication in analyzing massive datasets and identifying subtle patterns that might be missed by traditional methods [6].

The development of predictive models for type 2 diabetes (T2D) complications has been a process of continuous improvement. As these models have evolved, they have become powerful tools for doctors and patients to prevent or delay the devastating effects of diabetes complications, as highlighted in various literature reviews [6,7,8,9,10,11,12]. The goal is to create personalized prediction models that consider a patient’s medical history, allowing for the most effective preventative strategies for each individual.

The rationale behind designing and applying prediction models for T2D-related microvascular complications lies in their potential to lead to serious health problems if left unchecked. By predicting which patients are at higher risk, medical professionals can take proactive measures. Early interventions, such as tighter blood sugar control, lifestyle modifications, and medications can potentially prevent or delay the onset and progression of these complications. Prediction models also help to tailor personalized treatment plans to significantly improve patient outcomes. Furthermore, they can identify patients who would benefit most from intensive monitoring and specialist care, allowing for a more efficient allocation of healthcare resources [13].

The International Diabetes Federation (IDF) reported a global age-standardized prevalence of diabetes of 9.8% globally and 16.4% in the UAE among adults aged 20–79 years as of 2021 [13]. The most recent Global Burden of Disease (GBD) initiative reported the prevalence of T2D to be as high as 9.3% in the North Africa and Middle East (MENA) region [14]. The same study also projected that the MENA region would have a prevalence rate of 16.8% by 2050. As of 2021, Kuwait, Saudi Arabia, and the UAE have the highest prevalence of diabetes in the region [15]. These high rates of T2D in the region correlate positively with the rapid pace of economic development [15,16]. Therefore, understanding the risk factors associated with T2D microvascular complications, particularly those influenced by affluence, is crucial for effective management and prevention.

In this study, we applied supervised automated machine learning using the IBM SPSS Modeler package on two datasets and developed models for predicting microvascular complications in patients with T2D in Dubai, UAE. The first dataset, consisting of T2D patients with various comorbidities and complications, was used for model training. The second dataset, comprising an independent cohort of newly diagnosed T2D patients with no comorbidities or complications, was used for model testing and validation. The microvascular outcomes were used as target variables, while clinical variables obtained from routine electronic health records were used as independent variables. The following three different automated model scenarios were employed and compared: Full All-Variable Model, Univariate-Selected Model, and Backward Stepwise Logistic Regression Model. The results in this study were compared with recent machine learning studies predicting retinopathy [17,18,19,20], nephropathy [21,22,23,24], and peripheral neuropathy [25,26] in patients with T2D. The clinical utility of the prediction model outcomes and their potential impact on patient care decisions were also assessed [27]

## 2. Materials and Methods

### 2.1. Patients

The study was conducted at the health facilities of Dubai Health, an integrated academic health system recently assembled in Dubai, UAE. It comprises six tertiary care hospitals, 26 primary health centers (PHCs), Mohammed Bin Rashid University (MBRU), and the Al Jalila Research Foundation. In this study, we developed machine learning models to predict microvascular complications in T2D patients. Only consenting adults (18–80 years) testing negative for GAD antibodies (ELISA Test Kit; Demeditec Diagnostics, GmbH, Kiel, Germany) and diagnosed with T2D (fasting blood glucose (FBG) ≥125 mgdL, hemoglobin A1c (HbA1c) ≥6.5%) by a registered medical practitioner were included in the study. Patients with conditions causing secondary diabetes were excluded. All other types of diabetes (MODY and LADA) were also excluded. The training dataset was obtained from a cohort of 348 T2D patients with at least two co-morbidities (hypertension, hyperlipidemia, and obesity) or complications [28]. They were selected from a database of 620 patients randomly enrolled from two tertiary healthcare centers, Dubai Diabetes Center and Dubai Hospital, between January 2020 and December 2022. The average duration of T2D in these patients was 14 years. To externally test and validate the model, an independent cohort of 338 newly diagnosed T2D patients with complete data was selected from a database of 558 patients screened in PHCs between January 2022 and December 2023. The maximum duration of T2D recorded in this cohort was 2.5 years without comorbidities or T2D complications. The patients in both groups were undergoing treatment with different medications (metformin, thiazolidines, SGLT2 inhibitors, and GLP-1 agonists) prescribed in routine practice. For each patient, the clinical and laboratory data were obtained from the SALAMA electronic health record system used by all health facilities affiliated with Dubai Health.

Records of microvascular complications were obtained from patients’ notes in SALAMA Health Information System. The recorded medical history, comorbidities, and complications were reconfirmed via face-to-face interviews during revisits. For retinopathy, both proliferative or non-proliferative findings or laser treatment were accepted as evidence. For nephropathy, an eGFR <60 mL/min, or albuminuria of 30–300 mg/g was accepted as evidence. For neuropathy, records of pain in the calf muscle, foot ulcers, gangrene, amputations, or angioplasty were accepted as evidence.

Biochemical parameters, such as serum insulin, C-peptide, FBG, HbA1c, blood urea, estimated glomerular filtration rate (eGFR), high-density lipoprotein (HDL), low-density lipoprotein (LDL), triglycerides, total cholesterol, alanine aminotransferase (ALT), aspartate aminotransferase (AST), alkaline phosphatase (ALP), and urine creatinine, were measured at Dubai Hospital Laboratories using the fully automated Roche Cobas^®^ 6000 analyzer (Roche Diagnostics, Basel, Switzerland). A blood pressure > 120/80 mmHg, measured using a sphygmomanometer, was considered indicative of hypertension, while body mass index (BMI) was calculated by dividing a person’s weight (kg) by the square of their height in meters (kg/m^2^).

### 2.2. Statistics

The data were analyzed using IBM-SPSS for Windows (version 29.0; SPSS Inc., Chicago, IL, USA). Continuous variables (serum insulin, C-peptide, etc.) were described using measures of central tendency and dispersion or medians and interquartile ranges depending on the data distribution. Categorical variables, such as sex and hypertension status, are presented as frequencies and proportions. The Kolmogorov–Smirnov test was used to assess the normality of continuous variables. The Mann–Whitney U test was used to compare the risk of microvascular complications between cases and controls (microvascular (-/+)) if the distribution of variables was non-normal, whereas the independent t-test was used if the variables were normally distributed. The chi-square test was used to assess the dependency between categorical variables (sex and hypertension) and the risk of complications. A *p*-value of less than 0.05 was considered significant for all statistical analyses.

To identify predictors from significant variables in the new dataset, forward logistic regression was applied using microvascular complications as the dichotomous dependent variable as applied to the group of patients who were predicted as negative versus the group of patients who were predicted as positive (No = 0; Yes = 1) and all significantly different variables between the two groups as independent variables.

### 2.3. Study Design and Techniques

The IBM SPSS Modeler (version 18.0; IBM North America, New York, NY, USA) software package, designed for data science and machine learning tasks, based on deep learning and artificial neural networks, was used in this study for risk analysis and regression. This software has successfully been used in several studies [29,30,31,32].

#### 2.3.1. Selection of Predictors

Initially, machine learning (ML) was applied to the training data of 348 patients with long-term T2D for model selection. To predict microvascular risk in the 338 newly diagnosed T2D patients, a case-control design (+/-) was employed using three different automated model scenarios for selection of predictors:

Scenario 1 (Full All-Variable Model): The microvascular outcomes were used as the target variables, while all of the other 24 clinical variables in the training data set were used as independent variables for the new data set.

Scenario 2 (Univariate-Selected Model): Univariate analysis was used to select the significant clinical independent variables for predicting microvascular complications between cases and controls in the training data set. Only 16 significant variables were included as independent predictors for determining the target variables in the new data set.

Scenario 3 (Backward Stepwise Logistic Regression Model): A backward binary logistic model was employed to select the best set of predictors from the independent variables in the training data set. These predictors were then used to determine the target variables in the new data set.

#### 2.3.2. Prediction Models

The IBM Modeler simplifies predictive model building by eliminating the need for complex code. It assists throughout the model creation process, from automatic data cleaning to appropriate algorithm selection and model deployment. In our study, we applied SPSS Modeler to predict microvascular complications in newly diagnosed T2D patients at PHCs. By leveraging built-in algorithms and data visualization tools, the selected predictive models estimated a patient’s likelihood of developing microvascular complications.

The software explores 14 potential predictive models [C5.0 Decision Tree Algorithm (C5), Logistic Regression (LR), Bayesian Network (BN), Linear Discriminant Analysis (D), Linear Support Vector Machine (LSVM), Random Tree (RT), Extreme Gradient Boost Linear (XGBL), Extreme Gradient Boost Tree (XGBT), Chi-Square Automatic Interaction Detection (CHAID), Quick Unbiased Efficient Statistical Tree (QUEST), Classification and Regression Tree (C&R Tree), Neural Network (NN), Decision List (DL), and Tree AS (Tree AS)]. It sequentially explores all 14 models, automatically selects the best 5–6 models and issues their results.

In the training dataset, the study used the following 25 independent variables associated with long-duration T2D as predictors (serum insulin, C-peptide, sex, hypertension, body mass index (BMI), fasting blood glucose (FBG), HbA1c, blood urea, eGFR, high-density lipoprotein (HDL), low-density lipoprotein (LDL), triglycerides, total cholesterol, alanine aminotransferase (ALT), aspartate aminotransferase (AST), alkaline phosphatase (ALP), urine creatinine, albumin/creatinine ratio, platelet count, serum albumin, age at diagnosis, age at visit, and duration of T2D). Following extensive training, the model was primed to predict microvascular complications in the data set of the newly diagnosed T2D patients. The three microvascular complication variables (retinopathy, nephropathy, and peripheral neuropathy) were used as one target variable and was defined as a dichotomous variable if detected and referred to as [1], while [0] was assigned in the absence of all three target variables.

#### 2.3.3. Model Processing and Performance

The number and types of algorithms predicting target variables were determined by supervised machine learning (ML) using the Auto-Classifier algorithms in the IBM SPSS Modeler software. The performance of these models was evaluated, and the relative importance of each predictor was determined for each individual classifier to highlight the relative significance of each predictor in the model estimation. After the models were run, their performances were assessed using a confusion matrix and receiver operating characteristic curve (ROC). The confusion matrix was used to determine the number of true positives (TPs), true negatives (TNs), false positives (FPs), and false negatives (FNs). Accuracy, classification error, sensitivity, specificity, positive predictive value (PPV), and negative predictive value (NPV) were calculated. The ROC curve plots, true positive rate against the false negative rate at various threshold points, and area under the curve (AUC) were used to evaluate the discriminatory ability of the prediction models. Model prediction stability was assessed using the standard deviation of the accuracy of each model. A significance test was conducted to evaluate the differences between all the models using Cochran’s Q test. The McNemar test was applied to further compare the performances of the two models with best results.

## 3. Results

### 3.1. Characteristics of Clinical Data Sets

A comparison of the laboratory data of the 348 T2D patients with long-duration T2D in the presence of comorbidities and complications [Mean T2D duration of 14.4 (±8.1) years] that formed the training dataset and 338 newly diagnosed T2D patients [Mean T2D duration of ≤2.5 (±1.5) years] with no comorbidities or complications that formed the test dataset, is shown in Table 1. On comparing data from the two data sets, all clinical and laboratory variables were found to be statistically significant, except BMI. HDL, triglycerides, ALT, AST, and platelet counts were not significantly different between the two groups (Table 1).

Among the 348 patients in the training data set, 241 (69.3%) had microvascular complications. Retinopathy was detected in 117 patients (33.6%), nephropathy in 43 patients (12.4%), and peripheral neuropathy in 189 patients (54.3%). Clinical and laboratory variables in the training data set of patients with and without microvascular complications were compared. Nine variables were statistically different (Table 2).

### 3.2. Prediction Models

#### 3.2.1. Scenario 1 (Full All-Variable Model)

In Scenario 1, all 25 independent variables were used for training, while microvascular complications were used as the target variable. The Auto-Classifier ran 14 different models, and the five best models were selected for each target variable (Table 3).

The five algorithms that best predicted microvascular complications were BN, CHAID, C5, NN, and D model (Table 3). Among these, the Bayesian Network (BN) was identified as the best model for the following reasons: (i) The simplicity of the model compared to other ensemble models, utilizing all 25 variables involved in the prediction. (ii) The model exhibited stable performance with the smallest standard deviation of accuracy (0.47), showing no signs of overfitting or underfitting. (iii) It had a higher discriminative ability (AUC = 87.1%) and reasonable accuracy (67.82%). Additionally, the model’s sensitivity to detect microvascular complications was 173/195 (89%), and the specificity was 63/84 (75%).

**Figure 1:**
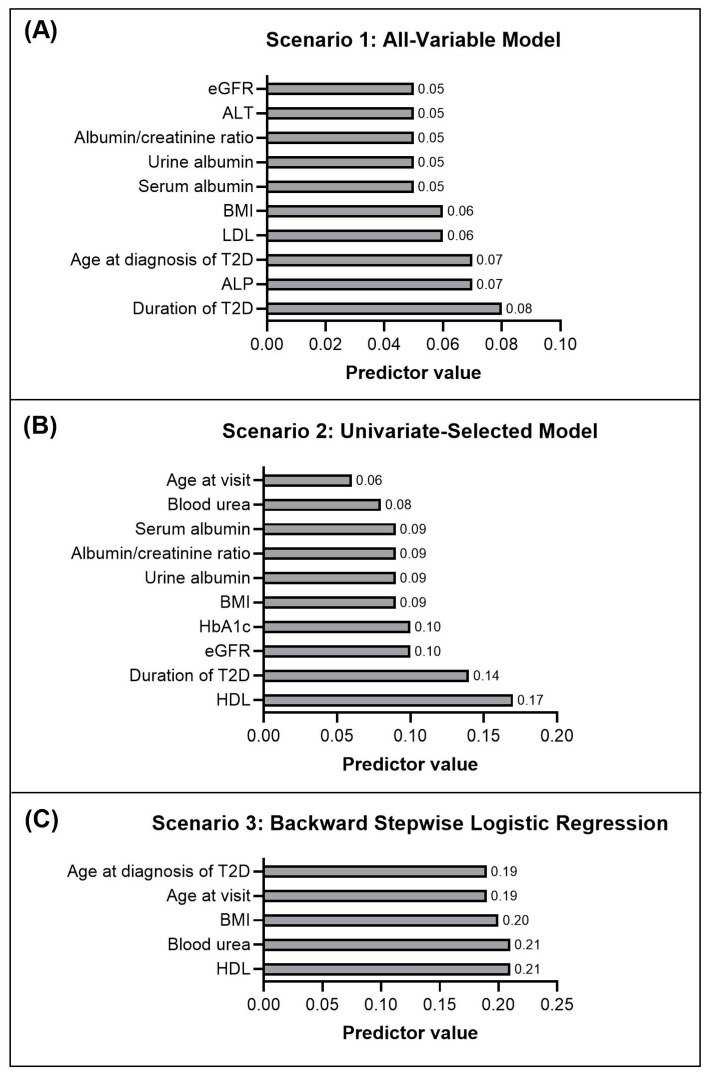
Predictor importance for the best models at predicting the microvascular complications for the three scenarios: Bayesian Network (BN) was predicted as the best model in Scenario 1 (**A**) and Scenario 2 (**B**); logistic regression was the best model for Scenario 3 (**C**). The predictors are shown on the *y*-axis and their values on the *x*-axis.

The duration of T2D was the most important predictor of combined microvascular complications, followed by ALP level and age at diagnosis (Figure 1A). Other important predictors included levels of LDL, BMI, serum albumin, urine albumin, albumin/creatinine ratio, ALT, and eGFR. In Scenario 1, the model predicted microvascular complications in 119 out of 338 patients, corresponding to a prevalence of 35.2% with a 95% confidence interval of 30.1–40.3%.

#### 3.2.2. Scenario 2 (Univariate-Selected Model)

In this scenario, the dependent variables were selected by univariate analysis from variables in the training data set. The univariate analysis’s goodness of fit was χ^2^ = 54.484, df = 5 with a *p*-value < 0.001. Eleven variables that showed statistical significance with a target variable, such as cases and controls within the training dataset, were selected: age at visit, duration of T2D, BMI, HbA1c, blood urea, serum creatinine, eGFR, HDL, urine albumin, albumin/creatinine ratio, and serum albumin.

The five best algorithms identified for predicting microvascular complications were the BN, LR, LSVM, CHAID, and DM models (Table 3). Among these, as in Scenario 1, the BN was identified as the best model since it has an accuracy of 74% associated with a small standard deviation (0.44). This indicates that it is a stable model with a high discriminative ability (AUC = 84.2%). Additionally, the model’s sensitivity to detect microvascular complications was 201/233 (86%), while its specificity was moderate at 55/104 (53%).

HDL was the most important predictor for microvascular complications, followed by the duration of T2D, eGFR, and HbA1c (Figure 1B). Other important predictors included urine albumin, serum albumin, albumin-to-creatinine ratio, blood urea and age at visit. When the node of microvascular Scenario 2 was used to predict microvascular outcomes in new diabetes patients, the model identified 113 out of 338 patients as predicted microvascular cases, corresponding to a prevalence of 33.8% with a 95% confidence interval of (28.4–38.5%).

#### 3.2.3. Scenario 3 (Backward Stepwise Logistic Regression Model)

Backward logistic regression was used to identify the best independent variables in the training dataset as predictors for the target variable of microvascular complications. The variables identified were BMI, blood urea, HDL, age at diagnosis, and age at visit. The five best algorithms identified for predicting microvascular complications in this scenario were the LR, BN, LSVM, NN, and DM models (Table 3). Among these, as in Scenario 1, BN was identified as the best model with an accuracy of 75%, associated with small standard deviation (0.43). This means it was a stable model with high discriminative ability (AUC = 77.3%). Additionally, the model’s sensitivity to detect microvascular complications was 217/241 (95%) and its specificity was 32/107 (31%).

As illustrated in Figure 1C, HDL was the most important predictor for microvascular complications, followed by blood urea, BMI, age at visit, and age at diagnosis, with the same rank of importance. When Scenario 3 was used to predict microvascular outcomes in newly diagnosed T2D patients, the model identified 134 out of 338 patients as predicted microvascular cases, corresponding to a prevalence of 39.4% with a 95% confidence interval of (34.4–44.9%).

### 3.3. Comparison Between Prediction Models

Table 3 illustrates the discriminative ability of the prediction models across the three scenarios. It was evident that the AUC in the first scenario was the highest at 0.871, while the second scenario produced an AUC of 0.842, and the third produced an AUC of 0.773. Although all three scenarios demonstrated discriminative ability, Scenarios 1 and 2 exhibited the best capability for discrimination.

Regarding the validation of case detection by complication, the predicted cases across the three scenarios showed the following distribution: 54 (16%) patients were predicted to be positive in one scenario, 42 (12.4%) patients were predicted to be positive in two scenarios, and 76 (22.5%) patients were predicted to be positive in all three scenarios, while 166 (49.1%) patients were predicted to be negative in all three scenarios (Supplementary Tables S1–S3).

### 3.4. Clinical Utility of the Prediction Models

All independent variables were compared between the two groups of the newly diagnosed T2D patients: 76 (22.5%) were predicted as positive in all three scenarios, while 166 (49.1%), were predicted negative in all three scenarios. Table 4 shows the clinical characteristics of the newly diagnosed T2D patients who are predicted to develop microvascular complications compared with those who are not predicted to develop complications using the three prediction scenarios.

#### 3.4.1. Statistical Analysis of Predictors

Forward logistic regression was applied to the clinical parameters of the 76 patients predicted as positive versus the 166 patients predicted as negative for all three scenario models. The microvascular complications were used as the dichotomous dependent variable (No = 0; Yes = 1) and all 16 statistically significant predictors as independent variables. The model ran in 12 steps and ended with the results shown in Table 5. The goodness of fit of the model is indicated by a chi-square value of 199.828, with five degrees of freedom and a *p*-value of less than 0.001. The R-squared value was 0.916, implying that 91.6% of the variation in microvascular measures is explained by BMI, HDL, adjusted for age at diagnosis of the disease, age, and urine albumin (Table 4).

#### 3.4.2. Probability of Risk of Microvascular Complications

To calculate the likelihood of microvascular complications in each new T2D patient the values of the five variables obtained from the forward logistic regression were used to derive the following formula:

$$\begin{array}{c} If;Logit\left(p\right)=\log(\frac{P}{1-P})=-8.653-0.524\;age\;at\;diagnosis+0.53\;Age\;at\;visit\;+\\ 0.833\;BMI-0.473\;HDL+0.04\;Urine\;Albumin\\ Then. \end{array}$$

$$\frac{P}{1-P}e^{\left(-8.653-0.524\;age\;at\;diagnosis+0.53\;Age\;at\;visit+0.833\;BMI-0.473\;HDL+0.04\;Urine\;Albumin\right)}\;$$

$$P=\frac{e^{\left(-8.653-0.524\;age\;at\;diagnosis+0.53\;Age\;at\;visit+0.833\;BMI-0.473\;HDL+0.04\;Urine\;Albumin\right)}}{1+e^{\left(-8.653-0.524\;age\;at\;diagnosis+0.53\;Age\;at\;visit+0.833\;BMI-0.473\;HDL+0.04\;Urine\;Albumin\right)}}$$

**Figure 2:**
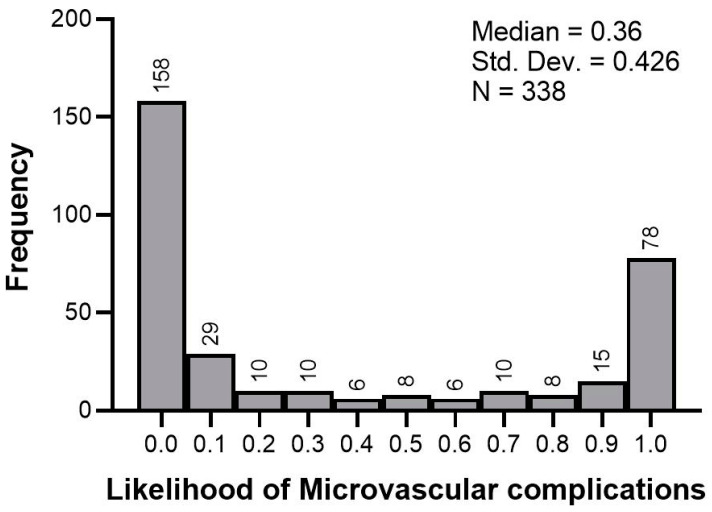
The frequency distribution of the likelihood of developing microvascular complications in newly diagnosed T2D patients.

The above equation was programmed in an Excel sheet in such a way that inputting a patient’s values for the five clinical variables, the probability of developing microvascular complications would be estimated. The distribution of the probability estimates in the 338 newly diagnosed T2D patients is displayed in Figure 2. Three risk categories are suggested according to the probability distribution: (i) no risk: 0.00–0.20 (n = 192); (ii) low risk: 0.21–0.80 (n = 50); and (iii) high risk: 0.81–1.00 (n = 96). The clinical characteristics of patients in different risk categories for developing microvascular complications is shown in Table 6.

## 4. Discussion

In this study, we employed supervised automated machine learning in IBM SPSS Modeler to develop models capable of predicting microvascular complications in patients with newly diagnosed type 2 diabetes (T2D) in Dubai, UAE. Two patient groups were recruited: a training set of 348 patients with various comorbidities and complications, and an independent testing set of 338 newly diagnosed patients without any comorbidities or complications. Using Modeler, we explored 14 potential predictive models, selecting the top 5–6 and prioritizing them in sequence. We evaluated the following three automated modeling scenarios: Full All-Variable Model (Scenario 1), Univariate-Selected Model (Scenario 2), and Backward Stepwise Logistic Regression Model (Scenario 3).

Patients in the training data set exhibited poor glycemic control, declining pancreatic and kidney functions, and various T2D complications. However, they also showed enhanced insulin sensitivity and favorable lipid profiles, likely because of intensive treatment.

Using IBM Modeler for automated prediction of T2D complications offered distinct advantages over comparing multiple individual models. The software automates model selection, training, and tuning across multiple algorithms, reducing the time and expertise required to independently run multiple models, each with different preprocessing, hyperparameter tuning, and optimization needs. IBM Modeler provides a centralized, streamlined evaluation process with built-in capabilities to assess models side by side, selecting the best one based on preset criteria like accuracy, sensitivity, and specificity. The platform also manages the entire workflow—from data preprocessing and feature selection to model evaluation—simplifying standardization and reducing the likelihood of errors that could arise with separate models.

By using a single platform, reproducibility and consistency are ensured, which is essential for clinical validation, as the process remains transparent and less fragmented. IBM Modeler’s ensemble learning automation further enhances accuracy by combining the strengths of multiple models, often outperforming individual models in predicting complex conditions like T2D complications.

The Bayesian Network (BN) emerged as the model with the highest discriminatory power and the most reliable algorithm for predicting microvascular complications across the three prediction scenarios, achieving an AUC of 77–87%, accuracy of 68–75%, sensitivity of 86–95%, and specificity of 53–75%. The prevalence of microvascular complications ranged from 34% to 39% across all scenarios, with BN’s performance aligning with some previous studies [3,4,5,6,7,8]. Variability in prior model performance likely results from differences in model types, predictor selection, and validation methods across studies [3,4,5,6,7,9].

Our study employed rigorous methodologies, building on prior research [3,4,5,6,7,8] while enhancing model performance by using 14 automatically ranked models in SPSS Modeler. We exclusively tested parameters from routine electronic health records, avoiding complex biomarkers not commonly used in clinical practice. To ensure external validity, we validated predictions on a cohort of newly diagnosed T2D patients from primary healthcare centers, avoiding sample-splitting techniques and using internal validation instead.

A subgroup of newly diagnosed T2D patients (n = 76, 22.5%) was consistently predicted as positive by all three model scenarios, while another subgroup (n = 166, 49.1%) was predicted as negative. Significant differences were observed between these subgroups in 16 clinical predictors. In two of the three model scenarios used, the duration of T2D was identified as a significant predictor of combined microvascular complications. Many of the 16 predictors identified (e.g., age at diagnosis and visit, BMI, eGFR, blood urea, urine albumin, HbA1c, and HDL) tend to worsen as the duration of diabetes increases. Yet, when further forward logistic regression was applied to these clinical parameters, the duration of T2D could not be validated as a predictor. The logistic regression analysis revealed that over 90% of the variation in microvascular measures could be explained by BMI, HDL, adjusted for age at diagnosis and visit, and urine albumin.

The predicted risk factors for microvascular complications (HDL, duration of T2D, eGFR, and HbA1c) were consistent with traditional T2D severity risk factors. However, they do not directly imply causation, except for early albuminuria predicting nephropathy [24]. To facilitate rapid and accurate T2D complication diagnosis, we developed an Excel formula that estimates the probability of microvascular complications based on the five identified clinical predictors, categorizing patients into the following three risk levels: no risk, medium risk, and high risk. Our findings highlight the importance of integrating machine learning tools into clinical practice to improve the diagnosis, prevention, and management of diabetes complications. In agreement with other studies [3,4,5,6,7,8,9], we found that machine learning-based models outperformed traditional regression models [18,19,20,22]. Although our prediction models were effective despite small cohort sizes, future studies should use larger cohorts from similar populations to validate and generalize these findings.

We were unable to assess the patient’s glucose homeostasis in detail, as the study relied on clinical data recorded in the electronic health record system (SALAMA) in Dubai, which did not include information on clinical interventions. Continuous glucose monitoring (CGM) devices, which measure glucose variability, are not part of routine practice in Dubai, so we were unable to incorporate glucose variability into our analysis. This also limited our assessment of insulin resistance to HOMA-IR, which is measured using fasting blood glucose and fasting insulin and is commonly used in clinical settings due to its simplicity and non-invasiveness. More robust and accurate methods, such as the Hyper-insulinemic Euglycemic Clamp (HEC) and the Frequently Sampled Intravenous Glucose Tolerance Test (FSIVGTT), were not accessible to us. The Quantitative Insulin Sensitivity Check Index (QUICKI), which also uses fasting blood glucose and insulin, has similar limitations to HOMA-IR in terms of accuracy. Therefore, only HOMA-IR was used as a measure of insulin resistance.

Another limitation of our study is that the predicted risk factors for microvascular complications (HDL, duration of T2D, eGFR, and HbA1c) were consistent with traditional T2D severity risk factors but did not directly imply causation, except for early albuminuria, which is a predictor of nephropathy [24]. To strengthen our findings the different forms of microvascular complications need to be assessed independently.

## 5. Conclusions

The Bayesian Network effectively predicted the risk of microvascular complications in newly diagnosed T2D patients in Dubai, demonstrating high accuracy, sensitivity, and specificity across different model scenarios. Key predictors, including BMI, HDL, age at diagnosis, age at visit, and urine albumin level, provide clinicians with valuable insights to tailor interventions and improve patient outcomes.

## Figures and Tables

**Table 1:** Demographic and laboratory data of T2D patients from primary care centers (without microvascular complications) compared with patients from tertiary care centers (with complications).

	**Patients Without Microvascular Complications** **[Test Data Set]** **(n = 338)**	**Patients with Microvascular Complications [Training Data Set]** **(n = 348)**	***p*-Value**
Sample Collected From	**Primary Healthcare Centers**	**Tertiary Healthcare Centers**	
	**Mean (±SD)**	**Mean (±SD)**	
Age at visit (years)	48.97 (9.6)	56.32 (10.9)	<0.001
Age at diagnosis (years)	46.5 (9.58)	41.91 (10.67)	<0.001
Duration of T2D (years)	2.48 (1.49)	14.42 (8.14)	<0.001
BMI at visit (Kg/m^2^)	31.58 (5.59)	31.27 (5.6)	0.436
FBG (mg/dL)	135.28 (46.43)	146.86 (52.47)	<0.001
HbA1c (%)	7.01 (1.33)	7.63 (1.72)	<0.001
Serum insulin (µIU/mL)	19.69 (12.82)	14.04 (10.67)	<0.001
Serum C-peptide (nmol/L)	1.13 (0.4)	0.94 (0.51)	<0.001
HOMA-IR	6.69 (5.29)	5.25 (4.78)	<0.001
HOMA-B	111.39 (94.83)	85.14 (111.22)	<0.001
Platelet count (10^9^/L)	265.53 (67.48)	260.05 (74)	0.100
Serum albumin (g/dL)	4.32 (0.36)	4.22 (0.36)	0.008
Blood urea (mg/dL)	24.79 (7.26)	31.43 (16.61)	<0.001
Serum creatinine (mg/dL)	0.68 (0.18)	0.79 (0.41)	<0.001
eGFR (mL/min)	108.71 (10.65)	95.82 (20.85)	<0.001
HDL (mg/dL)	47.09 (11.54)	47.26 (12.32)	0.947
LDL (mg/dL)	104.57 (39.62)	85.97 (34.09)	<0.001
Triglycerides (mg/dL)	131.95 (75.3)	137.5 ((79.73)	0.493
Total cholesterol (mg/dL)	179.13 (75.31)	159.98 (39.88)	<0.001
ALT (IU/L)	28.16 (18.87)	25.15 (21.39)	0.002
AST (IU/L)	23.1 (11.41)	23.54 (17.22)	0.743
ALP (IU/L)	84.50 (52.51)	79.05 (26.71)	0.096
Urine albumin (mg/L)	12.35 (22.01)	73.15 (302.77)	<0.001
Urine creatinine (mg/dL)	129.82 (74.41)	108.23 (78.11)	<0.001
Albumin/creatinine ratio (mg/g)	10.2 (14.35)	117.84 (551.05)	<0.001

T2D, type 2 diabetes; BMI, body mass index; FBG, fasting blood glucose; HbA1c, hemoglobin A1c; HOMA-IR, homeostatic model assessment for insulin resistance; HOMA-B, homeostatic model assessment of β-cell function; eGFR, estimated glomerular filtration rate; HDL, high-density lipoprotein; LDL, low-density lipoprotein; ALT, alanine aminotransferase; AST, aspartate aminotransferase; ALP, alkaline phosphatase. All values are presented as mean (±SD). *p*-Value was determined using *t*-tests and the Mann Whitney *U*-Test. *p* < 0.05-significant.

**Table 2:** Comparison of clinical and laboratory variables between T2D patients, in the training data set, with and without microvascular complications.

	Patients with Microvascular Complications (n = 241)	Patients Without Microvascular Complications (n = 107)	*p*-Value
	Mean (±SD)	Mean (±SD)	
Age at visit (years)	57.13 (10.14)	54.50 (11.35)	0.042
Age at diagnosis of T2D (years)	41.31 (10.51)	43.24 (10.97)	0.143
Duration of T2D (years)	15.82 (6.38)	11.25 (6.6)	<0.001
BMI at visit (kg/m^2^)	31.75 (5.8)	30.18 (5.34)	0.028
FBG (mg/dL)	146.62 (51.88)	147.39 (54.03)	1.000
HbA1c (%)	7.74 (1.78)	7.39 (1.58)	0.052
Insulin (µIU/mL)	13.85 (10.79)	14.46 (10.44)	0.383
C-peptide (nmol/L)	0.95(0.55)	0.92 (0.41)	0.914
HOMA-IR	5.19 (4.87)	5.39 (95.75)	0.471
HOMA-B	84.98 (117.62)	85.5 (95.75)	0.423
Platelet count (10^9^/L)	257.81 (74.82)	265.08 (72.21)	0.241
Serum albumin (g/dL)	4.2 (0.38)	4.29 (0.32)	0.010
Blood urea (mg/dL)	33.13 (18.91)	27.61 (8.47)	0.006
Serum creatinine (mg/dL)	0.83 (0.47)	0.69 (0.18)	0.003
eGFR (mL/min)	93.03 (22.47)	102.07 (14.97)	0.002
HDL (mg/dL)	45.95 (11.83)	50.19 (12.66)	0.003
LDL (mg/dL)	85.89 (35.01)	86.14 (32.08)	0.902
Triglycerides (mg/dL)	139.82 (80.23)	132.27 (78.73)	0.287
Total cholesterol (mg/dL)	159.13 (40.5)	161.89 (38.58)	0.427
ALT (IU/L)	24.94 (22.8)	25.62 (17.88)	0.313
AST (IU/L)	22.27 (13.93)	26.5 (22.94)	0.193
ALP (IU/L)	80.51 (29.23)	75.76 (19.59)	0.408
Urine albumin (mg/L)	92.07 (360.13)	30.75 (65.5)	0.047
Urine creatinine (mg/dL)	105.92 (77.33)	113.36 (79.95)	0.243
Albumin/creatinine ratio (mg/g)	154.41 (649.95)	28.7 (65.07)	0.014

T2D, type 2 diabetes; BMI, body mass index; FBG, fasting blood glucose; HbA1c, hemoglobin A1c; eGFR, estimated glomerular filtration rate; HDL, high-density lipoprotein; LDL, low-density lipoprotein; ALT, alanine aminotransferase; AST, aspartate aminotransferase; ALP, alkaline phosphatase; HOMA-IR, homeostatic model assessment for insulin resistance; HOMA-B, homeostatic model assessment of β-cell function. All values are presented as the mean (±standard deviation). *p*-Value was determined by the *t*-test and Mann–Whitney *U* Test. *p* < 0.05, significant.

**Table 3:** Summary of the IBM Modeler Auto-Classifier’s performances in predicting microvascular complications in newly diagnosed T2D patients using the Full All-Variable Scenario model, Univariant-Selected model, and Backward Stepwise Logistic Regression model; all 25 independent variables were used in the training data set, and validation was conducted on newly diagnosed T2D patients from the same population.

Full All-Variable Model														
**Models**	**No.**	**Profit %**	**No, Field**	**TP**	**TN**	**FP**	**FN**	**Accuracy (%)**	**SD**	**AUC**	**Sensitivity**	**Specificity**	**PPV**	**NPV**
BN	279	78	24	173	63	21	22	67.82	0.47	0.871	0.89	0.75	0.89	0.74
CHAID	348	56	9	178	90	17	63	77.01	0.42	0.835	0.74	0.84	0.91	0.59
C5	348	68	10	206	75	32	35	80.75	0.35	0.839	0.85	0.70	0.87	0.68
NN	279	85	24	177	35	49	18	60.92	0.49	0.765	0.91	0.42	0.78	0.66
DM	348	93	22	148	81	26	93	65.81	0.47	0.741	0.61	0.76	0.85	0.47
Univariant Selected Model														
BN	337	60	10	201	55	49	32	73.56	0.44	0.842	0.86	0.53	0.80	0.63
LR	337	87	10	213	30	74	20	69.83	0.46	0.719	0.91	0.29	0.74	0.60
LSVM	348	81	10	223	28	79	18	72.13	0.45	0.735	0.93	0.26	0.74	0.61
CHAID	348	62	6	185	76	31	56	75	0.43	0.785	0.77	0.71	0.86	0.58
DM	348	83	10	153	78	29	88	66.38	0.47	0.733	0.63	0.73	0.84	0.47
Backward Stepwise Logistic Regression Model														
LR	348	89	5	217	32	75	24	71.55	0.451	0.731	0.90	0.30	0.74	0.57
BN	348	86	5	229	74	33	12	75.29	0.431	0.773	0.95	0.69	0.87	0.86
LSVM	348	85	5	222	81	26	19	71.26	0.453	0.732	0.92	0.76	0.89	0.81
NN	348	90	5	217	77	75	30	71	0.454	0.741	0.88	0.51	0.74	0.72
DM	347	75	5	154	68	38	87	63.79	0.481	0.731	0.64	0.64	0.80	0.44

BN, Bayesian Network; CHAID, Chi-Squared Automatic Interaction Detection; C5, C5 Tree Algorithm; NN, Neural Network, DM, Discriminant Model; LR, Logistic Regression; LVSM, Linear Support Vector Machine; TP, true positive; TN, true negative; FP, false positive; FN, false negative; SD, standard deviation; AUC, area under the curve; PPV, positive predictive value; NPV, negative predictive value.

**Table 4:** Clinical characteristics of the newly diagnosed T2D patients who are predicted to develop microvascular complications compared with those who are not predicted to develop complications using the three prediction scenarios.

	Newly Diagnosed T2D Patients Predicted to Develop Microvascular Complications (n = 76)	Newly Diagnosed T2D Predicted Not to Develop Microvascular Complications (n = 166)	*p*-Value
	Mean (±SD)	Mean (±SD)	
Age at visit (years)	47.46 (9)	49.92 (9.31)	0.030
Age at diagnosis of T2D (years)	44.84 (8.87)	47.5 (9.44)	0.019
Duration of T2D (years)	2.62 (1.57)	2.42 (1.46)	0.331
BMI at visit (kg/m^2^)	35.91 (5.57)	28.98 (3.93)	<0.001
FBG (mg/dL)	143.03 (54.82)	131.11 (44.45)	0.103
HbA1c (%)	7.42 (1.44)	6.81(1.22)	<0.001
Insulin (µIU/mL)	23.7 (14)	17.12 (10.84_	<0.001
C-Peptide (nmol/L)	1.22 (0.41)	1.06 (0.35)	0.005
HOMA-IR	8.21 (5.13)	5.71 (4.83)	<0.001
HOMA-B	135.47 (117.87)	101.87 (86)	0.057
Platelet count (10^9^/L)	267.97 (64.96)	265.65 (63.49)	0.780
Serum albumin (g/dL)	4.28 (0.29)	4.35 (0.41)	0.198
Systolic BP (mmHg)	128.47 (13.81)	123.83 (13.05)	0.035
Diastolic BP (mmHg)	79.67 (9.79)	76.3 (9.48)	0.006
Blood urea (mg/dL)	25.64 (7.22)	24.12 (7.18)	0.117
Serum creatinine (mg/dL)	0.72 (0.17)	0.66 (0.18)	0.005
eGFR (mL/min)	107.86 (10.72)	109.07 (10.38)	0.593
HDL (mg/dL)	37.64 (7.81)	52.81 (10.97)	<0.001
LDL (mg/dL)	102.01 (34.36)	102.07 (38.4)	0.783
Triglycerides (mg/dL)	147.55 (79.66)	124.02 (79.59)	0.002
Total Cholesterol (mg/dL)	172.74 (37.09)	180.46 (44.46)	0.261
ALT (IU/L)	32.53 (27.75)	25.84 (17.93)	0.007
AST (IU/L)	23.32 (11.71)	24.56 (20.59)	0.949
ALP (IU/L)	83 (27.67)	80.5 (25.66)	0.741
Urine albumin (mg/L)	18.54 (37.93)	9.5 (10.49)	0.001
Urine creatinine (mg/dL)	151.9 (82.34)	115.27 (71.86)	0.003
Albumin/creatinine ratio (mg/g)	11.43 (20.68)	8.24 (11.05)	0.184

T2D, type 2 diabetes; BMI, body mass index; FBG, fasting blood glucose; HbA1c, hemoglobin A1c; BP, blood pressure; eGFR, estimated glomerular filtration rate; HDL, high-density lipoprotein; LDL, low-density lipoprotein; ALT, alanine aminotransferase; AST, aspartate aminotransferase; ALP, alkaline phosphatase; HOMA-IR, homeostatic model assessment for insulin resistance; HOMA-B, homeostatic model assessment of β-cell function. All values are presented as the mean (±standard deviation). *p*-Value was determined by the *t*-test and Mann–Whitney *U* test. *p* < 0.05, significant.

**Table 5:** Results of the forward logistic regression identifying the five best independent variables as predictors for the target variable of microvascular complications. The presence of complications is considered as the dichotomous dependent variable (No = 0; Yes = 1) and all the 16 statistically significant variables as independent variables.

							95% CI	
Variables	B	S.E.	Wald	df	*p*-Value	OR	Lower	Upper
Age at diagnosis (years)	−0.524	0.333	2.476	1	0.116	0.592	0.308	1.137
Age at visit (years)	0.530	0.332	2.551	1	0.110	1.699	0.887	3.255
BMI at visit (Kg/m^2^)	0.833	0.178	21.863	1	0.000	2.300	1.622	3.261
HDL (mg/dL)	−0.473	0.104	20.687	1	0.000	0.623	0.508	0.764
Urine albumin (mg/L)	0.040	0.024	2.667	1	0.102	1.040	0.992	1.091
Constant	−8.653	5.212	2.756	1	0.097	0.000		

BMI, body mass index; HDL, high-density lipoprotein; B, coefficients; S.E., standard error; df, degrees of freedom; OR, odds ratio; CI, 95% confidence interval.

**Table 6:** Clinical and laboratory characteristics of newly diagnosed T2D with three categories of risk for developing microvascular complications.

	No Risk (n = 192)	Moderate Risk (n = 50)	High Risk (n = 96)	*p*-Value
	Mean (±SD)	Mean (±SD)	Mean (±SD)	
Age at diagnosis of T2D (years)	47.9 (9.6)	46.2 (9.3)	43.8 (9.3)	0.001
Age at visit (years)	50.3 (9.5)	48.9 (9.2)	46.3 (9.5)	0.002
Duration of T2D (years)	2.4 (1.5)	2.7 (1.6)	2.5 (1.5)	0.620
BMI at visit (kg/m^2^)	28.7 (4)	32.6 (3.4)	36.8 (5.5)	<0.001
FBG (mg/dL)	131 (42.2)	133.1 (33.1)	144.9 (59)	0.220
HbA1c (%)	6.9 (1.3)	6.9 (1)	7.3 (1.5)	0.038
HOMA-IR	5.7 (4.7)	8.0 (6.4)	8.0 (5.4)	<0.001
HOMA-B	102.8 (86.5)	127.8 (102.5)	120 (105.1)	0.172
Platelet count (10^9^/L)	261.4 (62.3)	281.6 (89.1)	265.8 (62.3)	0.246
Serum albumin (g/dL)	4.4 (0.4)	4.2 (0.3)	4.3 (0.3)	0.015
Blood urea (mg/dL)	25.1(7.9)	25.5 (6.2)	23.8 (6.3)	0.279
Serum creatinine (mg/dL)	0.7 (0.2)	0.7 (0.2)	0.7 (0.2)	0.321
eGFR (mL/min)	108.3 (10.3)	108.1 (10.1)	109.8 (11.6)	0.286
HDL (mg/dL)	52 (11.4)	43.2 (5.8)	38.8 (8.3)	<0.001
LDL (mg/dL)	105.8 (40.7)	105.6 (37.8)	101.3 (38.4)	0.697
Triglycerides (mg/dL)	124.3 (75.3)	143.1 (70.6)	142.2 (76.7)	0.014
Total cholesterol (mg/dL)	183.1 (46.1)	179.7 (42.4)	170.2 (39.6)	0.082
ALT (IU/L)	26.6 (18)	28.4 (22.4)	33.9 (27.8)	0.023
AST (IU/L)	25.3 (19.3)	22.6 (9.3)	23.8 (13.5)	0.823
ALP (IU/L)	85.1 (65.3)	84.2 (25.7)	83.4 (25.3)	0.560
Urine albumin (mg/L)	9.6 (10.8)	8.4 (7)	18.3 (34.8)	<0.001
Urine creatinine (mg/dL)	114.8 (70.6)	143.7 (79)	151.6 (76.1)	<0.001
Albumin/creatinine ratio (mg/g)	8.2 (10.9)	7.4 (13.2)	9.9 (17.1)	0.265

T2D, Type 2 diabetes; HOMA-IR, homeostatic model assessment for insulin resistance; HOMA-B, homeostatic model assessment of β-cell function; BMI, body mass index; FBG, fasting blood glucose; HbA1c, hemoglobin A1c; eGFR, estimated glomerular filtration rate; HDL, high-density lipoprotein; LDL, low-density lipoprotein; ALT, alanine aminotransferase; AST, aspartate aminotransferase; ALP, alkaline phosphatase. All values are presented as the mean (±standard deviation). *p*-Value was determined by the *t*-test and Mann–Whitney *U* test. *p* < 0.05, significant.

## Data Availability

Anonymized primary databases are available upon request. However, not all confidential patient information can be shared.

## Supplementary Materials

The following supporting information can be downloaded at: https://www.mdpi.com/article/10.3390/jcm13237422/s1.
