# Supplementary material for: Risk of Microvascular Complications in Newly Diagnosed Type 2 Diabetes Patients Using Automated Machine Learning Prediction Models
Source: J Clin Med. 2024 Dec 5;13(23):7422. doi: 10.3390/jcm13237422 (PMC11642608; doi:10.3390/jcm13237422)
Supplement: Supplementary file 1 [file jcm-13-07422-s001.zip › jcm-3322327-supplementary.pdf]

Supplementary Material

Supplementary Table S1: Characteristics of individuals predicted with Microvascular complications by all three scenarios.

	**Combined Microvascular Complications**		
	**N**	**Mean**	**SD**
Serum Insulin (μIU/ml)	76	23.70	13.99
C-peptide (nmol/L)	76	1.22	0.41
BMI (kg/m^2)	76	35.91	5.57
Systolic BP (mmHg)	76	128.47	13.81
Diastolic BP (mmHg)	76	79.67	9.79
FBG (mg/dL)	76	143.03	54.82
HbA1c (%)	76	7.42	1.44
Blood urea (mg/dL)	75	25.64	7.22
Serum creatinine (mg/dL)	74	0.72	0.17
eGFR (mL/min)	74	107.86	10.72
HDL (mg/dL)	76	37.64	7.81
LDL (mg/dL)	76	102.01	34.36
Triglycerides (mg/dL)	76	147.55	79.66
Total cholesterol (mg/dL)	76	172.74	37.09
ALT (IU/L)	73	32.53	27.75
AST (IU/L)	34	23.32	11.71
ALP (IU/L)	71	83.00	27.67
Urine albumin (mg/L)	65	18.54	37.93
Urine creatinine (mg/dL)	65	151.90	82.34
Albumin/Creatinine Ratio (mg/g)	65	11.43	20.68
Platelet Count (x10^6)	76	267.97	64.96
Serum albumin (g/dL)	74	4.28	0.29
HOMA-IR	76	8.21	5.13
HOMA-B	76	135.47	117.87
Age at diagnosis of T2D (years)	76	44.84	8.87
Age at visit (years)	76	47.46	9.00
Duration of T2D (years)	76	2.62	1.57

N, Number of patients; SD, Standard deviation; BMI, body mass index; BP, blood pressure; FBG, fasting blood glucose; HbA1c, hemoglobin A1c; eGFR, estimated glomerular filtration rate; HDL, high-density lipoprotein; LDL, low-density lipoprotein; ALT, alanine aminotransferase; AST- aspartate aminotransferase; ALP, alkaline phosphatase; HOMA-IR, homeostatic model assessment for insulin resistance; HOMA-B, homeostatic model assessment of β-cell function; T2D, Type 2 diabetes mellitus.

Supplementary Table S2: Characteristics of individuals predicted with Microvascular complication by two scenarios.

	Combined Microvascular Complications		
	**N**	**Mean**	**SD**
Serum Insulin (µIU/ml)	42	20.55	11.19
C-peptide (nmol/L)	42	1.19	0.41
BMI (kg/m^2)	42	34.31	6.79
Systolic BP (mmHg)	42	129.67	19.05
Diastolic BP (mmHg)	42	79.62	12.35
FBG (mg/dL)	42	144.29	50.44
HbA1c (%)	42	7.24	1.51
Blood urea (mg/dL)	36	25.19	6.00
Serum creatinine (mg/dL)	42	0.72	0.21
eGFR (mL/min)	42	109.45	11.91
HDL (mg/dL)	42	42.43	8.67
LDL (mg/dL)	41	104.54	41.32
Triglycerides (mg/dL)	42	141.24	70.59
Total cholesterol (mg/dL)	41	175.17	41.95
ALT (IU/L)	38	36.92	27.63
AST (IU/L)	21	24.14	13.17
ALP (IU/L)	37	89.05	22.44
Urine albumin (mg/L)	32	11.34	10.26
Urine creatinine (mg/dL)	32	144.22	77.76
Albumin/Creatinine Ratio (mg/g)	31	6.61	4.64
Platelet Count (x10^6)	40	258.68	63.17
Serum albumin (g/dL)	40	4.27	0.28
HOMA-IR	42	7.39	5.18
HOMA-B	42	104.98	92.28
Age at diagnosis of T2D (years)	42	44.74	9.87
Age at visit (years)	42	47.00	9.85
Duration of T2D (years)	42	2.26	1.43

N, Number of patients; SD, Standard deviation; BMI, body mass index; BP, blood pressure; FBG, fasting blood glucose; HbA1c, hemoglobin A1c; eGFR, estimated glomerular filtration rate; HDL, high-density lipoprotein; LDL, low-density lipoprotein; ALT, alanine aminotransferase; AST- aspartate aminotransferase; ALP, alkaline phosphatase; HOMA-IR, homeostatic model assessment for insulin resistance; HOMA-B, homeostatic model assessment of β-cell function; T2D, Type 2 diabetes mellitus.

Supplementary Table S3: Characteristics of individuals predicted with Microvascular complication by one scenario.

	**Combined Microvascular Complications**		
	**N**	**Mean**	**SD**
Serum Insulin (µlU/ml)	54	21.24	16.11
C-peptide (nmol/L)	54	1.16	0.50
BMI (kg/m^2)	54	31.35	4.86
Systolic BP (mmHg)	54	127.26	13.06
Diastolic BP (mmHg)	54	77.61	9.78
FBG (mg/dL)	54	130.20	33.20
HbA1c (%)	54	6.88	1.23
Blood urea (mg/dL)	51	25.18	8.27
Serum creatinine (mg/dL)	53	0.67	0.16
eGFR (mL/min)	54	108.19	10.56
HDL (mg/dL)	50	46.62	8.72
LDL (mg/dL)	50	116.68	47.81
Triglycerides (mg/dL)	50	126.46	51.61
Total cholesterol (mg/dL)	50	187.72	53.28
ALT (IU/L)	50	26.70	16.11
AST (IU/L)	21	26.24	14.41
ALP (IU/L)	49	96.12	119.06
Urine albumin (mg/L)	38	10.45	12.70
Urine creatinine (mg/dL)	38	129.58	61.55
Albumin/Creatinine Ratio (mg/g)	38	6.61	8.04
Platelet Count (x10^6)	54	267.98	83.35
Serum albumin (g/dL)	51	4.29	0.31
HOMA-IR	54	7.05	6.33
HOMA-B	54	111.76	82.27
Age at diagnosis of T2D (years)	54	47.13	10.47
Age at visit (years)	54	49.74	10.80
Duration of T2D (years)	54	2.61	1.53

N, Number of patients; SD, Standard deviation; BMI, body mass index; BP, blood pressure; FBG, fasting blood glucose; HbA1c, hemoglobin A1c; eGFR, estimated glomerular filtration rate; HDL, high-density lipoprotein; LDL, low-density lipoprotein; ALT, alanine aminotransferase; AST- aspartate aminotransferase; ALP, alkaline phosphatase; HOMA-IR, homeostatic model assessment for insulin resistance; HOMA-B, homeostatic model assessment of β-cell function; T2D, Type 2 diabetes mellitus.
